# Deconvolution in Alzheimer’s disease

**DOI:** 10.4103/NRR.NRR-D-25-00793

**Published:** 2025-09-29

**Authors:** Sho Oasa, Marianne Schultzberg, Lars Terenius

**Affiliations:** Department of Clinical Neuroscience, Center for Molecular Medicine, Karolinska Institutet, Stockholm, Sweden; Department of Neurobiology, Care Sciences & Society, Division of Neurogeriatrics, Karolinska Institutet, Stockholm, Sweden

Alzheimer’s disease (AD) is the most common origin of sporadic dementia. Rare familial forms have identified a central role for toxicity based on aggregation of peptide fragments generated from amyloid precursor protein (APP), named amyloid-beta (Aβ), which exists in two common forms, Aβ_1–40_ (Aβ_40_) and Aβ_1–42_ (Aβ_42_). The latter is more neurotoxic. A common clinical biomarker measured in blood is the ratio Aβ_42_/Aβ_40_.

Basal mechanisms that lead to AD have been elaborated extensively. Several studies indicate that the intermediate-size aggregates of Aβ, including protofibrils, are the most neurotoxic forms. Another core pathology of AD is the hyperphosphorylation of tau protein leading to neurofibrillary tangles, and also relevant in the pathology of AD is the activation of microglia, which in an earlier phase may be protective and with subsequent exposure leads to neuroinflammation (Kinney et al., 2018). The continuous presence of amyloid seems to be overriding the capacity of microglial cells to eliminate amyloid and restore the tissue, thereby leading to a chronic inflammatory state. Microglia are derived from the mesoderm and migrate to the brain during development where they serve as immune cells of the brain, constantly monitoring the tissue for changes/injury/other stimuli upon which they respond by becoming activated and produce inflammatory cytokines such as interleukin-1 and tumor necrosis factor alpha, and contribute to clearance of the tissue from cell debris or toxic substances by phagocytosis. It has been shown that microglia are activated in response to Aβ peptide and respond with secretion of inflammatory cytokines and are also able to take up Aβ.

As part of the innate immune system, dendritic cells (DCs) initiate an immune response upon detection of pathogens or cellular damage. DCs are also derived from the mesoderm but generally reside in the periphery. A recent study using Mendelian randomization suggested a potential causal association between peripheral immune cells and the risk of AD (Zhang et al., 2025), and there are several studies indicating that DCs are indeed recruited to the brain in response to amyloid pathology in AD and localize to amyloid plaques. It has been shown that Aβ_42_ influences DC differentiation, such as by modulating these cells towards a proinflammatory direction (Ciaramella et al., 2009). Whether the decoy substances studied also affect DCs needs further investigation.

Normally, inflammation ends by a process called resolution of inflammation, mediated by certain bioactive lipid mediators, so-called specialized pro-resolving lipid mediators (SPMs). The levels of SPMs are reduced in AD brain tissue and cerebrospinal fluid, supporting this as a potentiating mechanism of the continuous inflammation. Experiments in AD models indicate that treatment with SPMs reduces microglial activation, including Aβ_42_-induced nuclear factor-κB and inflammasome activation and Aβ_42_-induction of major inflammatory gene pathways in human microglia-like cells (Wang et al., 2025).

It is clear that microglial function is important for the brain and provides a protective role, and studies have shown that hypoxia may compromise this. Upregulation of hypoxia-inducible factor 1 (HIF1) was found in Aβ plaque-associated microglia with higher levels than in microglia more distant from the plaques, and mitochondrial activity in microglia is increased upon exposure to Aβ, and HIF1 mediates the mitochondrial metabolism of microglia. Also, the levels of HIF1 mRNA and protein were increased in AD brain tissue and a hypoxia-prone region is characterized by the presence of nude (microglia-free) Aβ plaques and the absence of microglia correlates with increased periplaque p-TAU dystrophic neurites, providing a modifiable AD risk factor. However, the sequence of events remains to be clarified, i.e., whether Aβ plaques induce local hypoxia stimulating HIF1 in plaque-associated microglia, or whether a local hypoxia condition impairs microglial cells with high HIF1 expression, followed by the reduction in Aβ clearance and depositions. The decoy (NSC 16224) reduces Aβ aggregates as can be seen in both Thioflavin T and FRET-FCS analysis (Oasa et al., 2023). Thus, it would be of interest to investigate whether this decoy may improve local hypoxia condition, and/or affect HIF1 expression.

The presence of chaperone proteins that recognize peptide/protein misfolds and aggregates is also protective. Currently, there is clinical evidence that immunogenic activity against protofibril forms may slow disease progression, although side effects of immunotherapy are so prevalent that surveillance is necessary, and the treatment is expensive. Although on an individual basis, individual patients may respond, but on a sample basis, the statistics are not significant. A recent example (Bateman et al., 2025) shows that in a clinical population of individuals with dominantly inherited AD, gantenerumab delays onset of severe symptoms during 2 years of treatment and reduces biomarkers such as the Aβ_42/40_ ratio and radioligand uptake detected by positron emission tomography. A serious constraint with antibody treatment is the risk for side effects due to additional immunogenetic activity.

Early attempts to block amyloid precursor protein degradation have been largely unsuccessful due to side effects and have largely been disbanded due to the lack of selectivity of the degrading enzyme, β-secretase 1. Numerous attempts to block production of Aβ at the target site would be more favorable. In the early study, we found that a five amino acid epitope sequence, Aβ16–20 (KLVFF) could be used to interfere with the aggregation *in vitro*. There are a fair number of studies, showing that natural or modified peptides covering this epitope similarly were inhibitors under equilibrium conditions. However, given the very strong forces favoring aggregation of the natural peptides, it is very unlikely that they will be active *in vivo*. Other approaches may be more successful. A study using phage selection of random peptide libraries identified peptides interacting with aggregation sites, not necessarily matching linear segments of the Aβ peptide, and with no binding to amyloid precursor protein (Reyes-Ruiz et al., 2021). Another approach originated from an antibody identified in a patient with AD and subsequently tested clinically as aducanemab. A peptide based on a major epitope for Aβ binding was identified and shown to be effective in inhibition of Aβ aggregation (França et al., 2024).

Cellular processes that react against misfolded or aggregated peptide/protein aggregates are also being approached. Interactions can occur in primary or secondary stages of the aggregation process and serve to eliminate toxicity. Such processes may not be sequence-specific. A chaperone-like molecule, nucleobindin-1, was active against the aggregation of islet amyloid polypeptide. An analogous observation was recorded with antibodies raised against a conjugate (nucleobindin-1 islet amyloid polypeptide complex). These antibodies bound not only islet amyloid polypeptide but also aggregates of Aβ and α-synuclein (Bonito-Oliva et al., 2019), emphasizing that the cross-reaction may be due to the common conformation of the respective aggregates/protofibrils and a common cross-β sheet structure.

**Figure 1:**
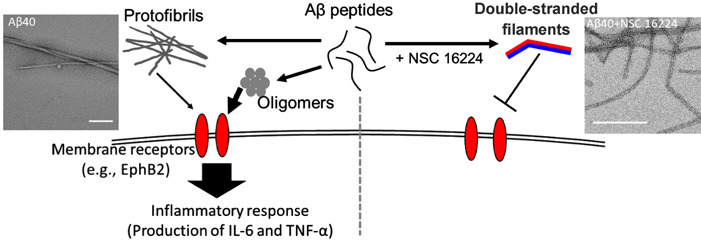
Schematic representation of the possible mechanisms of NSC 16224 decoy blocking. (Left) Aβ peptides form oligomers, protofibrils, and mature fibrils, as can be observed in the electron microscopy images. These forms activate membrane receptors such as EphB2 and transduce an inflammatory response via the productions of inflammatory cytokines. (Right) NSC 16224 generates double-stranded filaments, reducing the neurotoxicity. In addition, this unique structure did not trigger the inflammatory response, presumably due to a lack of recognition by membrane receptors. Microscopic images were reprinted with permission from Oasa et al. (2023). Aβ: Amyloid-beta; EphB2: ephrin type-B receptor 2; IL-6: interleukin-6; NSC: identifying number assigned by National Service Center at National Cancer Institute; TNF-α: tumor necrosis factor alpha.

Small molecules have also been found to eliminate neurotoxicity, which goes together with the change of conformation of aggregates. Such molecules have been named “decoys” of aggregation (Oasa et al., 2023). Decoys contributed to reducing Aβ aggregates and/or rewiring into non-toxic trajectories of the aggregation. When tested against microglia-like cell activation, most decoys did not significantly block Aβ-induced activation, with one exception (Oasa et al., 2024). The exceptional decoy (NSC 16224) both reduced aggregates and generated aggregates with a unique double-stranded structure, which apparently escaped the microglia radar (**Figure 1**). These observations give further substance to cross-reaction as based on aggregate structure rather than sequence homology.

The study also demonstrates that Aβ_42_ aggregates activate microglia-like cells to produce inflammatory cytokines at levels 20-fold higher than those induced by Aβ_40_ aggregates (Oasa et al., 2024). This suggests a greater role for Aβ_42_ in neuroinflammation and may also give an insight into the use of the classic biomarker Aβ_42_/Aβ_40_ in the clinic. One of the decoys, NSC 69318, has a quite deviant structure with dominant asymmetry and a large polycyclic domain (Oasa et al., 2023), showing selective reduction on the Aβ aggregation against the Aβ_42_, but it shows weak (but not statistically significant) activity with regard to both neurotoxicity and microglia-like cell activation. This may suggest that block of neurotoxicity and neuroinflammation can be differentiated.

It should be recognized that we have earlier reported that decoy activity can generate aggregates with very different topologies not observed without decoys, protofibril rods with buds and branches (NSC 9615 and NSC 26252) or even in a 2-dimensional sheet (NSC 100873). With the exceptional decoy, NSC 16224 (**Figure 1**), unique aggregates were observed which were not sensed by the microglia-like cells (Oasa et al., 2024). The decoy-induced aggregates are two-stranded, very compact, and apparently have a smooth surface that did not activate the microglia-like cells. With a block of neurotoxicity in SH-SY5Y cells and the release of pro-inflammatory cytokines related to microglia activation, NSC 16224 is obviously a strong candidate for further studies. Also relevant for further studies is the discovery that aggregates formed in the presence of NSC 69318 block neurotoxicity but are sensed by the microglia-like cells and with which effects of neuroinflammation alone would be accessible. Presumably, the conformation resulting from binding of this decoy peptide to Aβ_42_ still has the phenotype that is recognized by microglia and causes their activation. Aβ may bind to several receptors on microglia, including scavenger receptors, G-protein-coupled receptors, and Toll-like receptors (TLRs) (Yu and Ye, 2015). Interestingly, two of the G-protein-coupled receptors also bind SPMs, i.e., formyl peptide receptor 2 and chemokine-like receptor 1, which bind lipoxin A4, resolvin D1, and resolving E1, respectively. Chemokine-like receptor 1 (also called ChemR23) also binds the pro-inflammatory protein chemerin. These two receptors mediate uptake of Aβ, and formyl peptide receptor 2 also mediates an inflammatory response to Aβ. It would therefore seem as if compound NSC 69318 does not block the binding of Aβ_42_ to this receptor, nor that of the scavenger receptors CD36 or the receptor for advanced glycation end product, or toll-like receptors (TLR2, TLR4), also mediating inflammatory responses to Aβ (Yu and Ye, 2015). Further studies will be necessary to investigate which receptors compound NSC 16224 blocks to prevent inflammatory responses to Aβ_42_. Also, it would be of interest to investigate whether a combination of NSC 69318 blocking neurotoxicity and an SPM resolving inflammation would provide a potentiated effect on AD pathology and cognitive function *in vivo* in an animal AD model.

The data discussed here suggest that microglia may recognize three-dimensional topology that is not related to amino acid sequence. This ability may extend to B-cells and antibody production. One possible common mechanism is mechano-sensitive responsiveness, which has been reported in relation to tissue barriers (Maiques et al., 2025). There is also reason to reconsider the variance in neuropathology of AD, where for instance plaque formation is a common but not obligatory finding.

In summary, alterations in the Aβ aggregation trajectories by small molecule decoys can differentiate targets in AD.
